# Methylation Levels of *SLC23A2* and *NCOR2* Genes Correlate with Spinal Muscular Atrophy Severity

**DOI:** 10.1371/journal.pone.0121964

**Published:** 2015-03-30

**Authors:** Galina Yu. Zheleznyakova, Emil K. Nilsson, Anton V. Kiselev, Marianna A. Maretina, Lyudmila I. Tishchenko, Robert Fredriksson, Vladislav S. Baranov, Helgi B. Schiöth

**Affiliations:** 1 Department of Neuroscience, Uppsala University, Uppsala, Sweden; 2 Faculty of Biology, Saint-Petersburg State University, Saint-Petersburg, Russia; 3 Laboratory for Prenatal Diagnostics of Inherited Diseases, D.O. Ott Research Institute of Obstetrics and Gynecology RAMS, Saint-Petersburg, Russia; University of Edinburgh, UNITED KINGDOM

## Abstract

Spinal muscular atrophy (SMA) is a monogenic neurodegenerative disorder subdivided into four different types. Whole genome methylation analysis revealed 40 CpG sites associated with genes that are significantly differentially methylated between SMA patients and healthy individuals of the same age. To investigate the contribution of methylation changes to SMA severity, we compared the methylation level of found CpG sites, designed as “targets”, as well as the nearest CpG sites in regulatory regions of *ARHGAP22*, *CDK2AP1*, *CHML*, *NCOR2*, *SLC23A2* and *RPL9* in three groups of SMA patients. Of notable interest, compared to type I SMA male patients, the methylation level of a target CpG site and one nearby CpG site belonging to the 5’UTR of *SLC23A2* were significantly hypomethylated 19–22% in type III-IV patients. In contrast to type I SMA male patients, type III-IV patients demonstrated a 16% decrease in the methylation levels of a target CpG site, belonging to the 5’UTR of *NCOR2*. To conclude, this study validates the data of our previous study and confirms significant methylation changes in the *SLC23A2* and *NCOR2* regulatory regions correlates with SMA severity.

## Introduction

Proximal spinal muscular atrophy (SMA) is a monogenic disorder caused by degeneration of motor neurons in the anterior horns of the spinal cord [1]. SMA patients are subdivided into four types depending on age of onset and disease severity [2], [3]. The main genetic cause determining all types of SMA is a mutation in the survival motor neuron gene 1 (*SMN1*) encoding the SMN protein, which participates in snRNPs biogenesis [4]. SMN also has special functions in both motor neurons and muscles [5], [6], [7]. The copy number of the *SMN2* gene, centromeric copy of *SMN1*, is considered to be the main modifier of SMA severity [8], [9], [10]. Moreover, a connection of plastin 3 and profilin IIa proteins levels and a c.859G>C substitution in *SMN2* with SMA phenotype was identified [11], [12], [13]. However, the precise molecular mechanism of SMA pathogenesis is still unclear and the absence of an effective treatment has prompted a search for additional factors modifying SMA severity.

DNA methylation is an important epigenetic mechanism regulating gene expression in differentiated cells. DNA methylation profiles are partly determined during early embryogenesis, where changes in the DNA methylation profile might be initiated in response to different intrinsic and environmental factors [14], [15]. Aberrant DNA methylation was shown to be associated with various neurodevelopmental, neurodegenerative and psychiatric diseases, and is considered to be a biomarker of these pathological processes [16]. Interestingly, the expression of the *SMN2* gene in SMA patients is regulated by DNA methylation [17]. In a previous study, we carried out the first whole genome methylation analysis in SMA patients and compared those to healthy individuals of the same age. A strong difference in the methylation level of 40 CpG sites associated with different genes was revealed between SMA patients and healthy individuals [18]. The most significant CpG sites, belonging to the regulatory regions (promoter region, 5’UTR, 3’UTR) of *ARHGAP22*, *CHML*, and *SLC23A2*, are implicated in axonogenesis, cytoskeleton dynamics, neuronal development and maintenance. Other relevant findings were the changes in methylation levels of CpG sites related to *CDK2AP1* and *NCOR2*. Considering their function in chromatin remodulation and the connection with histone deacetylases, the main targets for SMA therapy [19]. Methylation alterations in promoter regions are mostly associated with gene expression levels [20], whereas 5’UTR and 3’UTR (untranslated regions) methylation levels might influence the elongation and termination of transcription [21].

The aim of the present study was to test if the methylation level of the CpG sites situated in regulatory regions of *ARHGAP22*, *CDK2AP1*, *CHML*, *NCOR2*, *SLC23A2* and *RPL9* that correlated with SMA severity. The CpG site which methylation level was compared previously between SMA patients and controls with whole genome methylation analysis is designated as “target”. We analyzed the methylation level of target CpG sites and some nearby CpG sites in the same amplicons, using bisulfite sequencing in SMA type I, II, III-IV patients’ groups. We also determined the expression levels of *ARHGAP22* and *NCOR2* genes in severe and mild SMA patients.

## Material and Methods

### Ethics statement

All healthy individuals, adult patients and parents of all children gave written informed consent to the diagnostic procedures. The analysis was approved by the ethics committee at D.O. Ott Research Institute of Obstetrics and Gynecology RAMS.

### Subjects

96 patients of different SMA types from the North-Western region of Russia were initially included in this study (Table 1). The information about SMA patients which were selected for gene expression analysis is presented in Table 2.

**Table 1:** SMA patients included in methylation analysis.

Type of SMA								
I (n = 24)			II (n = 43)			III-IV-asymptomatic (n = 29)		
**Sex**	**Mean age (months/SD)**	***SMN2* copy number frequency**	**Sex**	**Mean age (months/SD)**	***SMN2* copy number frequency**	**Sex**	**Mean age (years/SD)**	***SMN2* copy number frequency**
**F** 11	3.90/1.04	**2 *SMN2***–75%	**F** 23	35.72/7.35	**2 *SMN2***–9.3%	**F** 13	15.92/3.65	**2 *SMN2***–3.44%
**M** 13	6.05/1.7	**3 *SMN2***–20.83%	**M** 20	39.27/7.57	**3 *SMN2***–79.07%	**M** 16	18.50/2.83	**3 *SMN2***–48.28%
		**4 *SMN2***–4.17%			**4 *SMN2***–11.63%			**4 *SMN2***–44.83%
								**5 *SMN2***–3.45%

**Table 2:** SMA patients included in gene expression analysis.

Type of SMA			
I-II (n = 18)		III-IV-asymptomatic (n = 6)	
**Sex**	**Mean age (months/SD)**	**Sex**	**Mean age (years/SD)**
**F** 10	57.50/15.75	**F** 2	17.00/8.00
**M** 8	34.50/10.91	**M** 4	18.75 /4.96

### DNA extraction and bisulfite conversion of genomic DNA

Genomic DNA was isolated from peripheral blood leukocytes by phenol-chloroform extraction [22]. Bisulfite conversion was performed using Epitect 96 Bisulfite Kit (Qiagen) according to the manufacturer’s protocol. Bisulfite-treated DNA was amplified using EpiTect Whole Bisulfitome Kit 100 (Qiagen) to multiply template for multigene analysis.

### Quantitative DNA methylation analysis using bisulfite sequencing

**Fig 1:**
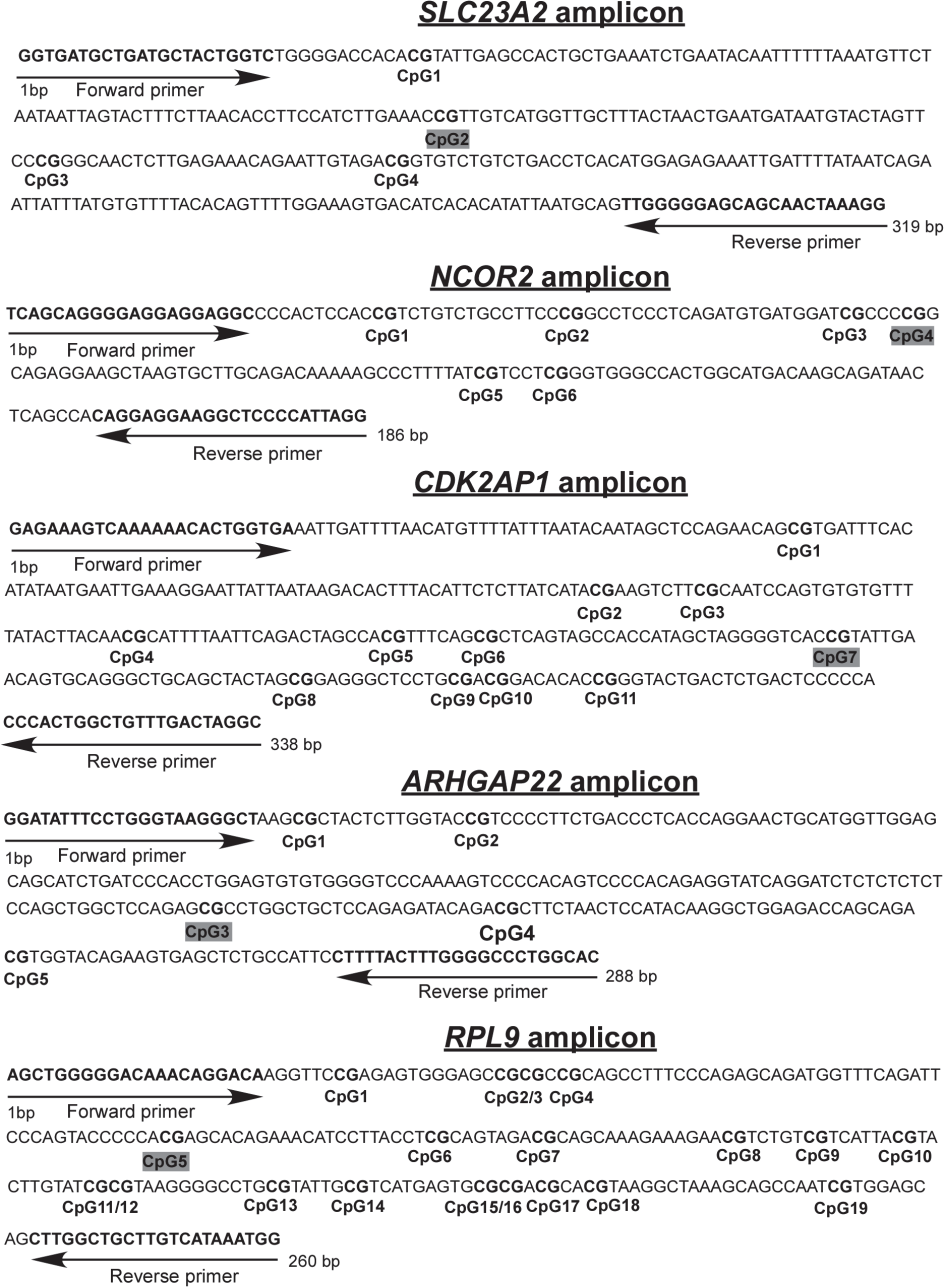
The sequence of analyzed amplicons with primers pairs’ and CpG sites’ positions. The target CpG site in each amplicon is highlighted.

Sequences of *ARHGAP22*, *CDK2AP1*, *CHML*, *NCOR2*, *SLC23A2*, and *RPL9* regulatory regions which included target CpG sites were obtained from UCSC Genome Browser database. Bisulfite sequencing primers were designed with Methyl Primer Express v1.0 (Applied Biosystems) so as the amplicons cover target CpG sites. All amplicons except of *CHML* also included a set of neighboring CpG sites. These regions were PCR amplified in duplicate from bisulfite-treated DNA. The primers’ sequences are presented in S1 Table. The information about sequence and length of the amplicons with CpG sites’ and primers’ positions are presented in Fig 1. Similarly PCR amplification efficiency for unmethylated and methylated fragments was controlled using Human Methylated and Non-methylated DNA Set (Zymo Research).

PCR reactions were performed in the final volume of 25 μl and contained 2.5 μl of bisulfite-treated DNA (30–70 ng/μl), 0.0625 μl of each primer (100 pmol/μl), 1 μl DMSO, 0.5 μl of SYBR Green I (1:50000; Invitrogen, Sweden) in TE buffer (pH 7.8), 0.25 μl of 25 mM dNTP mix (Fermentas), 2.5 μl 10× buffer, 4 μl of 25 mM MgCl2, 1 u of Hot Start Taq DNA polymerase (Thermo Scientific). Cycling conditions were as follows: 10 minutes initial denaturation step at 95°C, followed by 45 cycles of 95°C for 20 seconds, 30 seconds at optimal annealing temperature of primers (S1 Table), 30 seconds at 72°C, 5 minutes of final elongation at 72°C. Fluorescence was measured after the elongation phase. 81 cycles of 10 sec at 55°C with increasing increments of 0.5°C per cycle was performed for melting curve analysis. Bio-Rad iQ5 version 2.0 software (Bio-Rad Laboratories) was used to process real-time PCR data. Amplicons were purified using GeneJET PCR Purification Kit (Thermo Scientific).

DNA sequencing was performed using BigDye Terminator v3.1 Cycle Sequencing Kit (Applied Biosystems) on an ABI3730XL DNA Analyzer (Applied Biosystems) at Uppsala Genome Center. Cycle sequencing was as follows: 30 seconds initial denaturation step at 94°C, followed by 35 cycles of 94°C for 25 seconds, 50°C for 15 seconds, 60°C for 120 seconds. Each sample was sequenced twice. Amplification primers were used for sequencing, some loci were sequenced with forward and reverse primers, other loci were sequenced only with forward or reverse primer in duplicate. Data were analyzed with Sequence Scanner v.1.0 software and BISMA platform (Bisulfite Sequencing DNA Methylation Analysis) [23]. Samples with uncompleted bisulfate conversion and unsatisfied quality of sequencing were excluded on this step. The quantification of CpG sites’ methylation levels for all amplicons was performed using Epigenetic Sequencing Methylation analysis software [24]. The software was repeatedly used to determine the methylation profile of several genes [25], [26], [27]. The software algorithm allows analyzing the methylation percentage of each CpG site in amplicon without a cloning stage. However during the analysis CpG sites with bad quality of sequences, especially at the 3′ and 5′ ends of amplicons, were excluded. The exact number of SMA patients DNA samples analyzed for each CpG site of certain gene is presented below.

### Genotyping of *SLC23A2*, *CDK2AP1*, *RPL9* polymorphisms

Bisulfite sequencing analysis confirmed the existence of single nucleotide polymorphisms (SNPs) in CpG sites of interest: rs1279683 in target CpG site of *SLC23A2*, rs1109559 in target CpG site of *CDK2AP1* and rs2276890 in target CpG site of *RPL9*. Allele and genotypes distribution was determined.

### RNA isolation and cDNA synthesis

Peripheral blood leukocytes collection, storage of the collected cells, and RNA isolation were performed as described earlier [10]. cDNA synthesis was carried out with High Capacity RNA-to-cDNA Kit (Applied Biosystems) according to manufacture description.

### Gene expression analysis

The assay was carried out for *ARHGAP22* and *NCOR2* genes as earlier described. *GAPDH*, *H3b* and *ACTB* genes expression was used for normalization. The primers were designed with Beacon Primer Design 4.0 software (Premier Biosoft) and presented in S2 Table.

### Statistical analysis

Distribution normality for all variables was checked using Kolmogorov-Smirnov test. Because of non-Gaussian distribution, statistical comparisons of methylation levels among different groups of SMA patients were performed using the non-parametric Kruskal-Wallis test, although in the text and in the tables values are presented as mean ±SEM. To adjust for multiple comparisons we applied a Kruskal-Wallis permutation-based method, in which SMA phenotypes and observed methylation levels were shuffled 1000 times. The distribution of permutation-based _X_^2^ was used to determine _X_^2^ thresholds for a 95% confidence interval. Levene’s test was applied to compare a degree of variances between SMA groups. We included the methylation level of controls determined with the Infinium HumanMethylation450 BeadChip from our previous study [18]. However, the obtained data were not expected to be precisely matching our previous data, as methylation data generated by two different techniques might have discrepancy in values [28], [29]. The chi-square test was used to compare polymorphisms’ allele frequencies between SMA patients groups. Statistical analyses were performed using GraphPad Prism5 (GraphPad) and the statistical software R (www.r-project.org). A significance level of a = 0.05 or less was considered significant.

## Results

### Methylation levels of CpG sites located in the regulatory regions of *SLC23A2* and *NCOR2* genes correlate with SMA severity

The methylation level of four CpG (CpG1, CpG2, CpG3 and CpG4) sites was quantified in the 5’UTR of *SLC23A2*; CpG2 is the target site (Fig 1). This region is hypermethylated with a mean methylation level of >0.7 for SMA patients of all types (Table 3). A significant correlation between methylation levels and SMA severity was found for the CpG1 (_X_^2^ = 10.71, p = 0.005, Kruskal-Wallis test) and the CpG4 (_X_^2^ = 6.80, p = 0.03, Kruskal-Wallis test) sites (Table 3). We did not find significant correlation between the methylation levels of the CpG2 target site and SMA severity; it is important to note that the methylation levels of this site were lower in type III-IV SMA patients, compared to type I and type II SMA patients (Table 3).When splitting the analysis to include only males (n = 42) or females (n = 41), a significant correlation between methylation levels and SMA severity was found for the CpG1 (_X_^2^ = 6.80, p = 0.03, Kruskal-Wallis test) and the CpG2 target (_X_^2^ = 7.04, p = 0.03, Kruskal-Wallis test) sites among males and for CpG1 site (_X_^2^ = 7.21, p = 0.03, Kruskal-Wallis test) among females SMA patients (Table 3).

**Table 3:** Methylation levels (%) of CpG sites in the analyzed region of 5’UTR of *SLC23A2*.

	Type I			Type II			Type III-IV			Kruskal-Wallis			Levene’s Test		^ψ^Controls
CpG site	Mean	SEM	N	Mean	SEM	N	Mean	SEM	N	_X_^2^	Permuted _X_^2^	P-value	F	P-value	Mean
1	0.77	0.03	18	0.73	0.02	40	0.60	0.04	24	10.71	6.15	0.005	1.47	0.24	
2*	0.82	0.04	17	0.78	0.04	36	0.65	0.06	18	4.13		0.13	1.15	0.32	0.865
3	0.86	0.02	18	0.90	0.01	41	0.85	0.04	25	2.23		0.33	2.27	0.11	
4	0.86	0.03	18	0.77	0.03	40	0.72	0.04	25	6.80	5.52	0.03	0.57	0.57	
**Males**															
1	0.72	0.06	10	0.73	0.03	18	0.53	0.06	14	6.80	6.11	0.03	0.98	0.39	
2*	0.83	0.06	9	0.87	0.04	17	0.61	0.09	10	7.04	5.98	0.03	2.33	0.11	0.865
3	0.84	0.04	10	0.89	0.02	18	0.82	0.06	14	0.92		0.63	1.08	0.35	
4	0.82	0.04	10	0.76	0.05	17	0.64	0.07	14	4.90		0.09	0.57	0.57	
**Females**															
1	0.84	0.03	8	0.73	0.03	22	0.70	0.04	10	7.21	5.84	0.03	0.74	0.49	
2*	0.79	0.08	6	0.70	0.06	19	0.71	0.07	8	0.96		0.62	0.91	0.41	
3	0.89	0.03	8	0.91	0.01	23	0.89	0.03	11	0.92		0.63	1.79	0.18	
4	0.90	0.03	8	0.77	0.04	23	0.82	0.03	11	4.42		0.11	0.69	0.51	

*—*target CpG site*.

^ψ —^control values are from the previous study [18].

The methylation levels of four CpG sites, denoted CpG2 to CpG5, were analyzed within the 5’UTR region of *NCOR2* (Fig 1), where CpG4 is the target site. This region was hypermethylated in SMA patients of all groups (mean methylation level >0.7) (Table 4). A significant correlation between SMA severity and methylation levels was found for the CpG5 site (_X_^2^ = 6.23, p = 0.04, Kruskal-Wallis test). When performing methylation level analysis of male and female separately, a significant difference in the methylation levels was revealed for the CpG4 target site (_X_^2^ = 12.04, p = 0.002, Kruskal-Wallis test) among male SMA patients. No significant differences were observed between the methylation levels of any CpG sites among female SMA patients.

**Table 4:** Methylation levels (%) of CpG sites in the analyzed region of 5’UTR of *NCOR2*.

	Type I			Type II			Type III-IV			Kruskal-Wallis			Levene’s Test		^ψ^Controls
CpGsite	Mean	SEM	N	Mean	SEM	N	Mean	SEM	N	_X_^2^	Permuted _X_^2^	P-value	F	P-value	Mean
2	0.77	0.05	12	0.76	0.02	34	0.71	0.04	22	1.44		0.49	0.46	0.63	
3	0.94	0.01	13	0.94	0.01	34	0.89	0.04	22	0.38		0.83	2.68	0.76	
4*	0.84	0.03	11	0.80	0.02	34	0.73	0.04	22	5.32		0.07	0.85	0.43	0.79
5	0.89	0.05	9	0.80	0.03	29	0.70	0.05	19	6.23	5.90	0.04	1.85	0.17	
**Males**															
2	0.75	0.11	4	0.80	0.02	13	0.67	0.06	13	4.4		0.11	1.03	0.372	
3	0.93	0.02	5	0.95	0.01	13	0.85	0.06	13	2.98		0.22	1.60	0.22	
4*	0.83	0.05	4	0.84	0.01	13	0.67	0.06	13	12.04	5.23	0.002	1.64	0.21	0.79
5	0.91	0.04	3	0.83	0.04	13	0.67	0.08	11	5.15		0.08	1.12	0.34	
**Females**															
2	0.79	0.05	8	0.74	0.03	21	0.78	0.05	9	0.93		0.63	0.67	0.52	
3	0.95	0.02	8	0.93	0.01	21	0.94	0.03	9	1.6		0.45	0.55	0.58	
4*	0.84	0.05	7	0.78	0.02	21	0.83	0.02	9	4.65		0.98	0.65	0.53	
5	0.88	0.07	6	0.77	0.04	16	0.74	0.1	8	2.89		0.24	1.50	0.24	

*—*target CpG site*.

^ψ —^control values are from the previous study [18].

### Methylation levels of CpG sites located within the regulatory regions of *ARHGAP22*, *CDK2AP1*, *CHML* and *RPL9* genes

The methylation levels of eleven CpG sites were determined in a region located 1735–1398 bp upstream of the *CDK2AP1* TSS; CpG7 is the target site (Fig 1). The region demonstrated hypermethylation in all SMA patients’ groups (mean methylation level ≥0.9). The number of samples was not sufficient to properly compare methylation levels of the CpG7 target site between SMA patient groups because of the presence of the polymorphic rs1109559 (g.G>A) site disrupting the CpG7 site. The CpG7 site showed a 20–25% lower methylation level comparing to all other sites (Table 5). We did not reveal any significant difference in methylation level of any site among SMA patients of different types.

**Table 5:** Methylation levels (%) of CpG sites in a region located 1735–1398 bp upstream of *CDK2AP1* TSS.

	Type I			Type II			Type III-IV			Kruskal-Wallis		Levene’s Test		^ψ^Controls
CpG site	Mean	SEM	N	Mean	SEM	N	Mean	SEM	N	_X_^2^	P-value	F	P-value	Mean
1	0.94	0.01	16	0.86	0.06	20	0.94	0.02	19	1.81	0.41	1.65	0.2	
2	0.95	0.01	16	0.93	0.01	23	0.89	0.04	20	1.54	0.46	1.12	0.36	
3	0.90	0.02	16	0.92	0.01	24	0.94	0.01	20	4.37	0.11	1.78	0.18	
4	0.95	0.01	15	0.92	0.03	24	0.94	0.02	20	0.47	0.79	0.55	0.58	
5	0.86	0.02	16	0.84	0.04	24	0.84	0.02	20	0.66	0.72	0.36	0.70	
6	0.94	0.02	16	0.93	0.01	23	0.96	0.01	20	3.1	0.21	0.69	0.51	
7*	0.65	0.06	4	0.59	0.05	7	0.66	0.09	5	0.56	0.75	0.59	0.57	0.39
8	0.96	0.01	16	0.97	0.01	24	0.98	0.01	19	1.76	0.41	1.41	0.25	
9	0.90	0.02	11	0.93	0.01	21	0.94	0.02	17	5.68	0.06	0.86	0.16	
10	0.96	0.01	16	0.94	0.01	24	0.92	0.02	19	2.16	0.34	3.96	0.03	
11	0.94	0.02	14	0.95	0.01	24	0.95	0.01	20	0.06	0.97	0.38	0.69	

*—*target CpG site*.

^ψ —^control values are from the previous study [18].

The target CpG site located 1500 bp upstream of the *CHML* TSS was hypermethylated, with an average methylation level of >0.8 in all SMA patient groups. We did not detect a significant correlation between the CpG site methylation levels and SMA severity (type I SMA: 0.80 ± 0.04, N = 22; type II SMA: 0.85 ± 0.03, N = 35; type III-IV SMA: 0.87 ± 0.04, N = 24).

The methylation levels of two CpG sites (CpG3 and CpG4) were robustly determined in the 3’UTR region of *ARHGAP22*; CpG3 is the target site (Fig 1). CpG3 displayed a highly heterogeneous character of methylation between individuals, although the mean variation was similar for all three groups (S1 Fig). While CpG4 methylation levels were much less variable between different samples and the mean value was nearly similar for the patients of all three groups (S1 Fig). We did not find any difference in the methylation level of any sites between SMA patient groups.

The region located 1591–1333 bp upstream of the *RPL9* TSS contained 19 CpG sites, CpG5 is target site (Fig 1) and was shown to be highly hypomethylated in all SMA patients’ groups (average methylation level <0.1). An association between the methylation level and SMA severity was not found.

### Genotyping of single nucleotide polymorphisms (SNPs)

The distribution of allele and genotypes of SNPs which were found in target CpG sites linked with *SLC23A2*, *CDK2AP1* and *RPL9* genes is presented in Table 6. A difference in the alleles’ frequency of the rs1279683 polymorphic site was found between SMA patients of different types (_X_^2^ = 6.71, df = 2, p = 0.035, Chi-square test) (S2 Fig).

**Table 6:** Allele and genotype frequency of SNPs rs1279683, rs1109559 and rs2276890 in SMA patients.

*rs1279683(g.G>A) Target CpG2 site of 5’UTR of ***SLC23A2***	**type I (n = 22)**	**type II (n = 40)**	**type III-IV (n = 25)**
	%A = 18.18	%A = 20	%A = 38
	%G = 81.82	%G = 80	%G = 62
	AA = 1, AG = 6, GG = 15	AA = 1,AG = 14, GG = 25	AA = 4,AG = 11, GG = 10
rs1109559 (g.G>A) Target CpG7 site of regulatory region of ***CDK2AP1***	**type I (n = 16)**	**type II (n = 25)**	**type III-IV (n = 19)**
	%A = 71.88	%A = 30	%A = 68.42
	%G = 28.12	%G = 70	%G = 31.58
	AA = 9, AG = 5, GG = 2	AA = 11,AG = 13, GG = 1	AA = 9, AG = 8, GG = 2
rs2276890 (g.C>G) Target CpG5 site of ***RPL9***	**type I (n = 24)**	**type II (n = 32)**	**type III-IV (n = 28)**
	%C = 62.5	%C = 59.38	%C = 69.64
	%G = 37.5	%G = 40.62	%G = 30.36
	GG = 4,CG = 10, CC = 10	GG = 7,CG = 12, CC = 13	GG = 3,CG = 10, CC = 15

*—*Significant difference was found in the alleles’ frequency of the rs1279683 polymorphic site*.

### Relative expression levels of *ARHGAP22* and *NCOR2*

It was also meaningful to assess the expression of genes chosen for the methylation level analysis. Relative expression analysis was performed for *ARHGAP22* and *NCOR2* because of insufficient amount of biomaterial and low expression levels of some genes in blood cells. Highly interindividual variations were observed in the relative expression levels of both *NCOR2* and *ARHGAP22* independently of disease severity (S3 Fig). No significant difference was revealed in the expression levels of genes between SMA patients with severe (I-II) and middle (III-IV) forms.

## Discussion

In this study we tested the correlation between methylation levels and expression levels of *ARHGAP22*, *CDK2AP1*, *CHML*, *NCOR2*, *SLC23A2* and *RPL9* genes previously revealed after whole genome methylation analysis and SMA severity. Significantly decreased methylation levels of the CpG site in the 5’UTR of *SLC23A2* was previously identified in SMA male patients during whole genome methylation analysis [18]. *SLC23A2* encodes a SLC23A2 protein, a sodium/ascorbate co-transporter which provides high ascorbate concentration in the CNS. Ascorbate has several functions which are critical for the CNS [30]. Here we showed that the methylation level of two CpG1 and CpG4 sites in 5’ UTR of *SLC23A2* was significantly lowered by 14–17% in type III-IV versus type I SMA patients. Additionally, only in SMA males the methylation level of the CpG2 target and nearby CpG1 sites was lower by 19–22% in type III-IV versus type I SMA patients. As it was demonstrated, *SLC23A2* expression is dependent on the methylation levels of the promoter region [31]. The analysis employing the Encyclopedia of DNA Elements (ENCODE) data showed that the analyzed region is located close to the cluster of transcription factor binding sites and overlaps with signals for DNAse I hypersensitivity and histone modifications (H3K4me1, H3K4me3), implying an active regulatory structure (S4 Fig). Therefore lower methylation levels in type III-IV compared to type I SMA patients might suggest higher *SLC23A2* expression level.

Moreover, we found a significant increase in allele A frequency of the polymorphism rs1279683 (g.G>A), a polymorphic site located in the CpG2 target site, for the type III-IV SMA patients (S2 Fig). The G>A substitution disrupting the CpG site may lead to a decrease in DNA methylation of nearby CpG sites [32]. The number of samples was not sufficient to compare polymorphism rs1279683 genotype frequency between SMA patients of different types. However, it should be noted that the frequency of the AA genotype in type III-IV SMA patients is higher than in type I-II patients (S2 Fig). Both geographically matched healthy population and larger SMA patients’ cohort should be tested in the future for polymorphism rs1279683 allele and genotype frequency to make reliable conclusion about the correlation between this polymorphism frequency and SMA phenotype.

Comparison between type III-IV and type I SMA male patients demonstrated a 16% decrease in methylation levels of the CpG4 target site belonging to the 5’UTR of *NCOR2*. This is consistent with the data of whole genome methylation analysis that identified lower methylation levels of the CpG4 target site in healthy individuals [18]. It might be concluded that the methylation level of these sites in type III-IV SMA patients is rather similar to the methylation level of healthy individuals. Our results are also in good concordance with ENCODE project data showing hypermethylation of the CpG target site in all cell types (S5 Fig). Interestingly, these CpG sites overlap with signals for DNAse I hypersensitivity and histone modifications (H3K4me1, H3K27ac, H3K36me3), thus indicating an active regulatory region. We did not reveal any significant difference in the expression level of *NCOR2* between SMA patients of various types. This could be explained by small cohort size and unequal amounts of samples from type I and type III-IV SMA patients. Another explanation could be that *NCOR2* expression levels might be regulated independently of regulatory regions DNA methylation, involving other epigenetic mechanisms. Such microRNAs can influence *NCOR2* expression regulation [33].

No significant correlation was found between methylation levels of the CpG7 target site, located 1500 bp upstream of the *CDK2AP1* TSS, and SMA severity. However, it is important to note that methylation levels of this site were by 20–25% lower, when comparing to methylation levels of nearby CpG sites (Table 5). This could imply specific biological importance of this individual CpG site, as interaction with methyl-CpG-binding protein or methylation-sensitive transcription factor [34], [35]. According to ENCODE data the target CpG site is heterogeneously methylated, while nearby CpG sites are hypermethylated in cells of various types (S6 Fig). These regions also overlap with signals of histone modifications (H3K4me1, H3K4me3, H3K27ac, H3K36me3), indicating a structure of active chromatin.

*NCOR2* and *CDK2AP1* genes play an important role in transcription regulation. *NCOR2* encodes a SMRT protein (silencing mediator for retinoid and thyroid hormone receptor) [36]. SMRT, together with the NCoR1 protein, forms a core of multisubunit complexes that contain one of three different classes of histone deacetylases (HDAC) and consequently repress transcription of different genes [37]. It is interesting to note that the SMRT-NCoR complex is connected with HDAC1 and HDAC2 through mSin3A/B co-repressors [38]. It was demonstrated that mSin3A is associated with the SMN protein [39]. The *CDK2AP1* gene product, CDK2AP1 protein, is a subunit of the NuRD (Nucleosome Remodeling Deacetylase) complex, contained the histone deacetylases HDAC1, HDAC2 and the methyl-CpG-binding domain proteins MBD2 or MBD3 [40]. In the light of SMA interactions between SMRT and CDK2AP1 with histone deacetylases seems to be interesting, taking in the account that the main agents tested for SMA therapy are histone deacetylase inhibitors [41], [42].

The region upstream of the *RPL9* TSS was strongly hypomethylated in all SMA patient groups, although no difference was found in methylation level among different SMA types. This correlates with whole genome methylation analysis data showing a highly decreased methylation level of target CpG site in SMA patients comparing to healthy individuals [18]. *RPL9* DNA methylation alteration may be related to changes in different directions in the level of other ribosomal proteins which were described in SMA mice [43]. Alzheimer’s disease was also characterized by alterations in ribosomal proteins, rRNAs, and ribosomes, along with decreases in some protein factors stabilizing methylation [44]. Our finding of highly decreased methylation levels in *RPL9* could be additional evidence of significant methylation disturbances attending neurodegenerative disorders.

We did not observe significant differences in the methylation levels of CpG sites close to the TSS of the *CHML* gene among SMA patients. However a trend towards to an increase in methylation levels connected with mild SMA severity was observed. This is in accordance with our previous data on the whole genome methylation analysis showed significantly hypermethylated level for controls compared to SMA patients [18].

Whole genome methylation analysis showed that, CpG site located within the 3’UTR region of *ARHGAP22* was hypomethylated in healthy controls compared to intermediate methylation for SMA patients. Highly variable methylation levels of CpG sites for all analyzed groups in this study suggest different expression levels in SMA patients, but this is not obviously correlated with SMA severity.

To conclude, in this study we validated our previous results on whole genome methylation analysis in independent cohort of SMA patients. The results of this study confirm that methylation changes in the regulatory regions of *SLC23A2* and *NCOR2* are associated with SMA severity. Taking into account the data from ENCODE visualization, we suspect that DNA methylation changes might lead to expression changes of these genes. However, we could not define a difference in the expression level of *NCOR2* between severe and mild types of SMA patients. Thus, further studies are needed to access if DNA methylation changes of these genes are meaningful for their transcriptional regulation in patients with different SMA types. The methylation changes in *ARHGAP22*, *CDK2AP1*, *CHML* and *RPL9* were not detected among SMA patients of different types. However, it is not excluded that the difference between SMA patients and controls, indicated in our previous study, is meaningful for SMA pathogenesis. Our findings are limited to DNA methylation analysis in leukocytes. It should be taking in account that DNA methylation in leukocytes might not reflect the methylation level of the same genes in the motor neurons. Therefore our findings might require further confirmation by investigating methylation and expression levels of *SLC23A2* and *NCOR2* genes in disease-related tissue. However, we believe that analysis of methylation status in blood leukocytes is valuable in case of SMA as it is the most accessible biological material and could potentially be used in clinical practice for precise SMA severity detection and as a biomarker during SMA pharmacotherapy.

## Supporting Information

S1 Table Primers used in bisulfite sequencing analysis. (DOCX)

S2 Table Primers used in gene expression analysis. (DOCX)

S1 Fig The methylation level of two CpG sites located in 3'UTR of *ARHGAP22* in patients with different types of SMA. The vertical lines are the standard errors of the means.
(TIF)

S2 Fig Allele and genotype frequency of SNP rs1279683 (g.G>A) located in target CpG2 site of 5’UTR of *SLC23A2* in patients with different types of SMA. *—significant diference in allele frequency between SMA patients of different types (_X_^2^ = 6.71, df = 2, p = 0.035, Chi-square test).
(TIF)

S3 Fig *NCOR2* (A) and *ARHGAP22* (B) genes expression level in SMA patients with severe (I-II) and mild forms (III-IV). The vertical lines are the standard errors of the means.
(TIF)

S4 Fig Methylation and chromatin modification status within and in the proximity of 5’UTR *SLC23A2* analyzed region. DNA and chromatin modifications from ENCODE data are visualized with UCSC genome browser. Approximate borders of analyzed region are marked by red lines. The target CpG2 site (cg01326421) is hypermethylated in HepG2 (human liver hepatocellular carcinoma cell line), partially methylated in GM12878 (lymphoblastoid cell line) and K562 (human chronic myelogenous leukemia cell-line), unmethylated in HeLa-S3 (Human epithelial carcinoma cell line), HUVEC (human umbilical vein endothelial cells) and H1-hESC (H1 human embryonic stem cells). Analyzed region overlaps with signals for DNAseI hypersensitivity and mono- and triple-methylation of lysine 4 on histone H3 (H3K4me1, H3K4me3). In close proximity to analyzed region a cluster of transcription binding sites is situated.
(PDF)

S5 Fig Methylation and chromatin modification status within and in the proximity of 5’UTR *NCOR2* analyzed region. DNA and chromatin modifications from ENCODE data are visualized with UCSC genome browser. Approximate borders of analyzed region are marked by red lines. Target CpG4 site (cg 10996589) is hypermethylated in all cell types. The analyzed region overlaps with signals for DNAseI hypersensitivity and histone modifications (H3K4me1, H3K36me3, H3K27ac). Upstream of the analyzed region a cluster of transcription binding sites is situated.
(PDF)

S6 Fig Methylation and chromatin modification status within and in the proximity of the region located 1735–1398 bp of TSS of *CDK2AP1*. DNA and chromatin modifications from ENCODE data are visualized with UCSC genome browser. Approximate borders of analyzed region are marked by red lines. Target CpG7 site (cg09084244) is hypermethylated in GM12878, HeLa-S3 and HUVEC cell lines, partially methylated in H1-hESC and HepG2 cell lines, unmethylated in K562 cell line. Analyzed region overlaps with signals for DNAseI hypersensitivity and mono- and triple-methylation of lysine 4 on histone H3 (H3K4me1, H3K4me3). In close proximity to the analyzed region a cluster of transcription binding sites is situated.
(PDF)
