# Supplementary material for: Methylation Levels of SLC23A2 and NCOR2 Genes Correlate with Spinal Muscular Atrophy Severity
Source: PLoS One. 2015 Mar 30;10(3):e0121964. doi: 10.1371/journal.pone.0121964 (PMC4378931; doi:10.1371/journal.pone.0121964)
Supplement: S1 Table — (DOCX) [file pone.0121964.s001.docx]

**S1 Table.** Primers used in bisulfite sequencing analysis

| **Genomic position of analyzed amplicons** | **Primers’ sequence** | **T_A_, °C** |
| --- | --- | --- |
| ***SLC23A2* amplicon**  (5’UTR of *SLC23A2*)  chr20:4982973-4983291 | F 5'-GGTGATGTTGATGTTATTGGTT-3'  R 5'-CCTTTAATTACTACTCCCCCAA-3' | 63.3 |
| ***CHML* amplicon**  (1605-1410 bp upstream of TSS of *CHML)*  chr1:241800219-241800413 | F 5'-GATTAATATTGTTTGTTTTTATTTTTG-3'  R 5'-CTTTAACCTAAAAACTTTCAAATAAA-3' | 57.1 |
| ***RPL9* amplicon**  (1591-1333 bp upstream of TSS of *RPL9)*  chr4:39460399-39460658 | F 5'-AGTTGGGGGATAAATAGGATA-3'  R 5'-CCATTTATAACAAACAACCAAA-3' | 57.1 |
| ***CDK2AP1* amplicon**  (1735-1398 bp upstream of TSS of *CDK2AP1)*  chr12:123757625-123757962 | F 5'-GAGAAAGTTAAAAAATATTGGTGA-3'  R 5'-ACCTAATCAAACAACCAATAAA-3' | 58.0 |
| ***NCOR2* amplicon**  (5’UTR of *NCOR2)*  [chr12:125017059-125017244](http://genome.ucsc.edu/cgi-bin/hgTracks?hgsid=372962881_AtOpUnJLRnZdnKvxJmxJ1V3FavVB&db=hg19&position=chr12:125017059-125017244&hgPcrResult=pack) | F 5'-TTAGTAGGGGAGGAGGAGGT-3'  R 5'-CCTAATAAAAAACCTTCCTCCTA- 3' | 60.5 |
| ***ARHGAP22* amplicon**  (3’UTR of *ARHGAP22)*  chr10:49654166-49654453 | F 5'-GGATATTTTTTGGGTAAGGGTT-3'  R 5'-ATACCAAAACCCCAAAATAAAAA-3' | 61.4 |
