# Supplementary material for: Methylation Levels of SLC23A2 and NCOR2 Genes Correlate with Spinal Muscular Atrophy Severity
Source: PLoS One. 2015 Mar 30;10(3):e0121964. doi: 10.1371/journal.pone.0121964 (PMC4378931; doi:10.1371/journal.pone.0121964)
Supplement: S2 Table — (DOCX) [file pone.0121964.s002.docx]

**S2 Table.** Primers used in gene expression analysis

| **Gene** | **Primers’ sequence** | **T_A_, °C** |
| --- | --- | --- |
| *NCOR2* | F 5’-TCAAAATAAACCAGGCGATG-3’  R 5’-CTCCACCTTCTTCTCCCA-3’ | 58.4 |
| *ARHGAP22* | F 5’-GAAGAAGCAGAGGAGCAT-3’  R 5’-CCAGGAGGAAGTTCAGTC-3' | 61.5 |
| *GAPDH* | F 5’-CGCCAGCCGAGCCACATC-3’  R 5’-CGCCCAATACGACCAAATCCG-3’ | 55.4 |
| *ACTB* | F 5’-GATCGGCGGCTCCATCCT-3’  R 5’-GACTCGTCATACTCCTGCTTGC-3’ | 60.5 |
| *H3b* | F 5’-ATCCGCCGCTACCAAAAG-3’  R 5’-CGAAGATCGGTCTTGAAGTC-3’ | 57.6 |
