# Supplementary figures and images for: Methylation Levels of SLC23A2 and NCOR2 Genes Correlate with Spinal Muscular Atrophy Severity
Source: PLoS One. 2015 Mar 30;10(3):e0121964. doi: 10.1371/journal.pone.0121964 (PMC4378931; doi:10.1371/journal.pone.0121964)

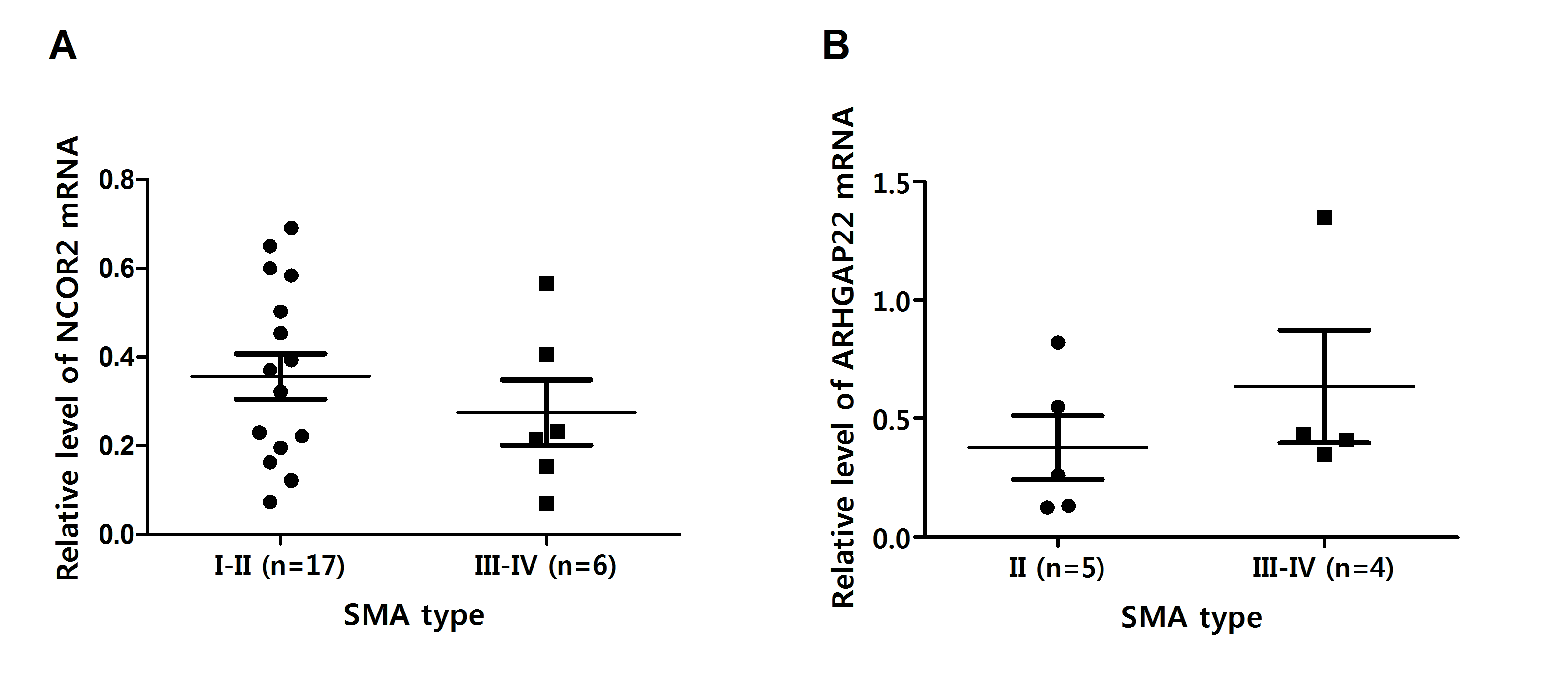

Supplement: S3 Fig — The vertical lines are the standard errors of the means. (TIF) [file pone.0121964.s005.tif]

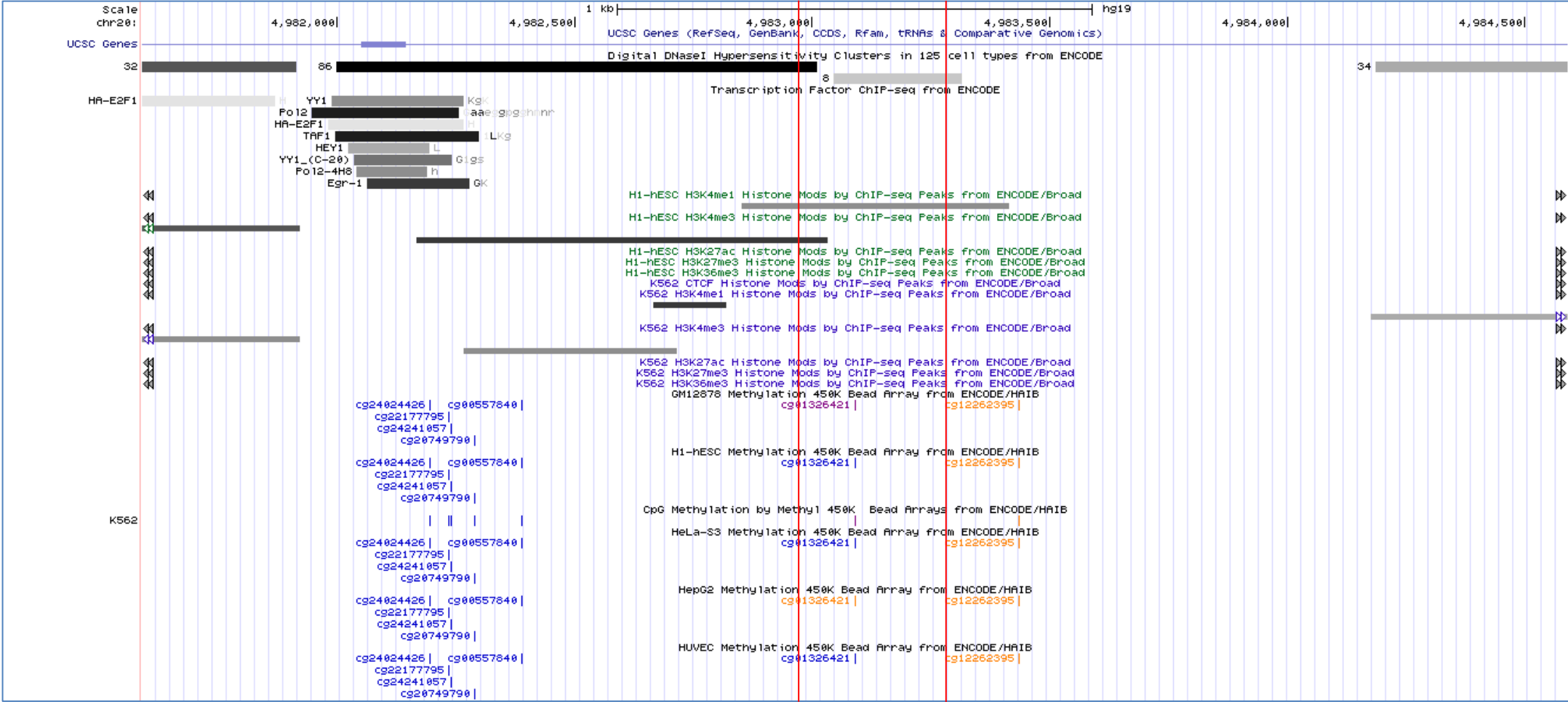

Supplement: S4 Fig — DNA and chromatin modifications from ENCODE data are visualized with UCSC genome browser. Approximate borders of analyzed region are marked by red lines. The target CpG2 site (cg01326421) is hypermethylated in HepG2 (human liver hepatocellular carcinoma cell line), partially methylated in GM12878 (lymphoblastoid cell line) and K562 (human chronic myelogenous leukemia cell-line), unmethylated in HeLa-S3 (Human epithelial carcinoma cell line), HUVEC (human umbilical vein endothelial cells) and H1-hESC (H1 human embryonic stem cells). Analyzed region overlaps with signals for DNAseI hypersensitivity and mono- and triple-methylation of lysine 4 on histone H3 (H3K4me1, H3K4me3). In close proximity to analyzed region a cluster of transcription binding sites is situated. (PDF) [file pone.0121964.s006.pdf]

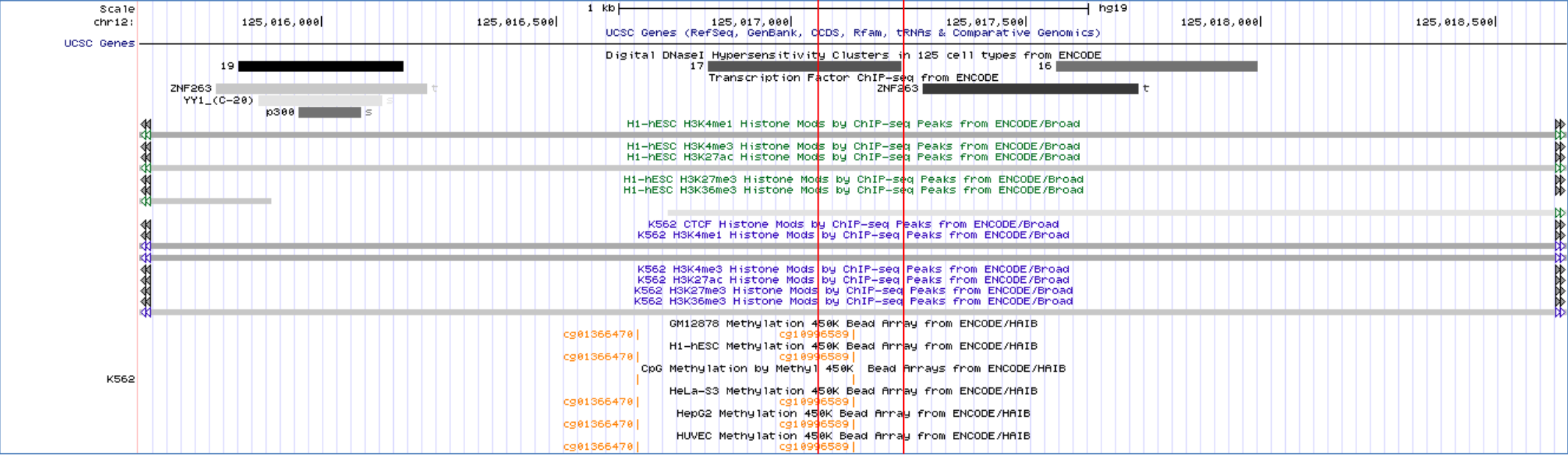

Supplement: S5 Fig — DNA and chromatin modifications from ENCODE data are visualized with UCSC genome browser. Approximate borders of analyzed region are marked by red lines. Target CpG4 site (cg 10996589) is hypermethylated in all cell types. The analyzed region overlaps with signals for DNAseI hypersensitivity and histone modifications (H3K4me1, H3K36me3, H3K27ac). Upstream of the analyzed region a cluster of transcription binding sites is situated. (PDF) [file pone.0121964.s007.pdf]

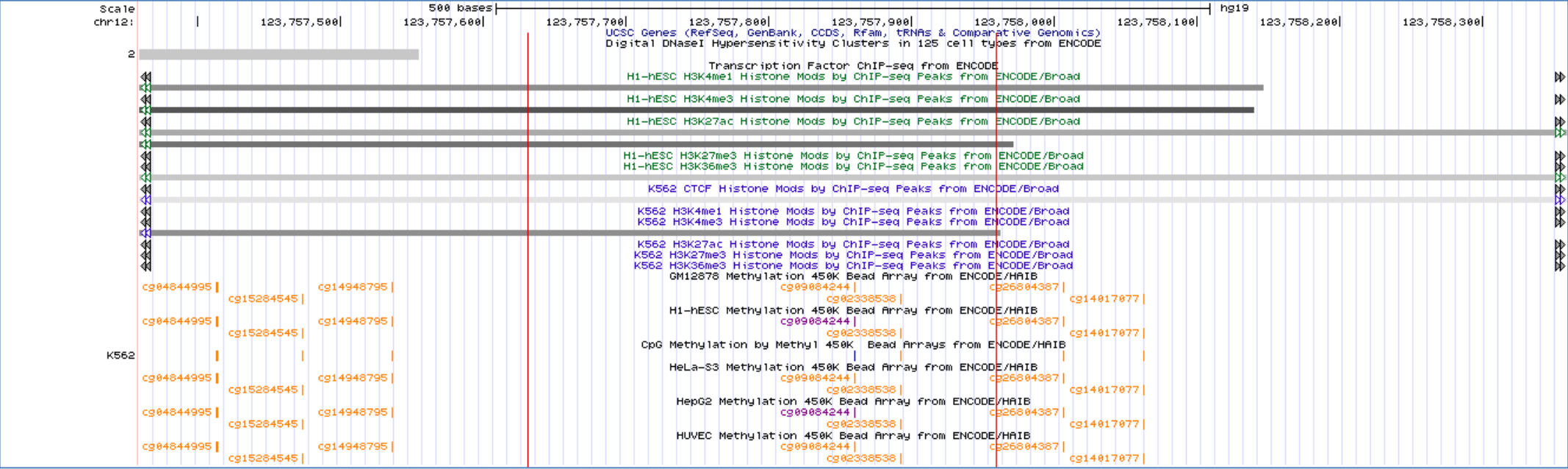

Supplement: S6 Fig — DNA and chromatin modifications from ENCODE data are visualized with UCSC genome browser. Approximate borders of analyzed region are marked by red lines. Target CpG7 site (cg09084244) is hypermethylated in GM12878, HeLa-S3 and HUVEC cell lines, partially methylated in H1-hESC and HepG2 cell lines, unmethylated in K562 cell line. Analyzed region overlaps with signals for DNAseI hypersensitivity and mono- and triple-methylation of lysine 4 on histone H3 (H3K4me1, H3K4me3). In close proximity to the analyzed region a cluster of transcription binding sites is situated. (PDF) [file pone.0121964.s008.pdf]
